# Supplementary material for: Molecular understanding of label-free second harmonic imaging of microtubules
Source: Nat Commun. 2019 Aug 6;10:3530. doi: 10.1038/s41467-019-11463-8 (PMC6684603; doi:10.1038/s41467-019-11463-8)
Supplement: Supplementary file 1 — Supplementary Information [file 41467_2019_11463_MOESM1_ESM.pdf]

Supplementary information

Molecular understanding of label-free second harmonic imaging of microtubules

Van Steenbergen et al.

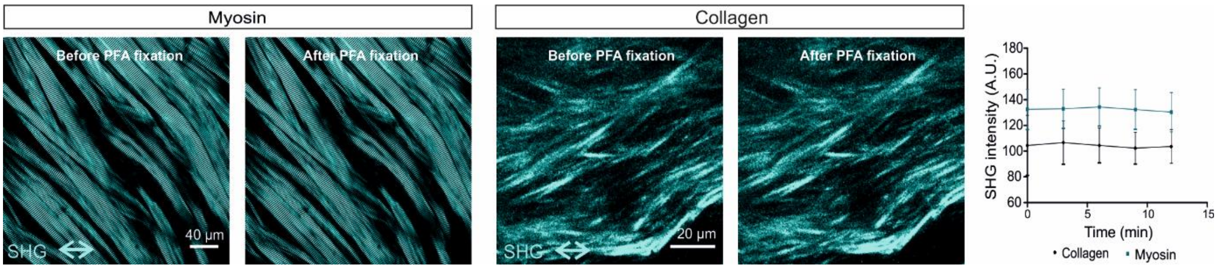

Supplementary Figure 1.

SH from myosin and collagen is preserved upon paraformaldehyde fixation.

Myosin from striated muscle in the mouse esophagus before and after fixation with paraformaldehyde (PFA, 4%) shows no difference in intensity (n=3 regions; p>0.05 when comparing SH signal intensity at t=0 min and t=12 min Mann Whitney test). Similar SH signal intensities were also recorded in collagen fibers from mouse tail tendon before and after PFA (4%) fixation (n=3 ; p>0.05 when comparing SH signal intensity at t=0 min and t=12 min Mann Whitney test). Data presented as means ± standard error of the mean. All source data are provided as a Source Data file.

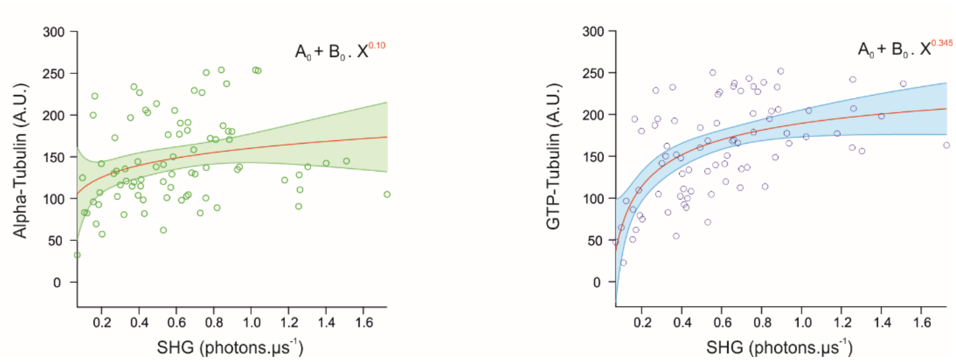

Supplementary Figure 2.

A power fit through GTP-tubulin is closer to the theoretical power relation with SH

A power fit was plotted through the data points from Figure 3A (relation of α-tubulin and SH signal intensity, green data) and 5A (GTP-bound tubulin dimer and SH signal intensity, blue data) using Igor Pro 8 (Wavemetrics). The resulting power fit (red line) for GTP-bound tubulin dimers (0.345) is closer to the theoretical 0.5 power relation with SH rather than the α-tubulin data (0.10). Confidence intervals are marked as color shaded area. All source data are provided as a Source Data file.

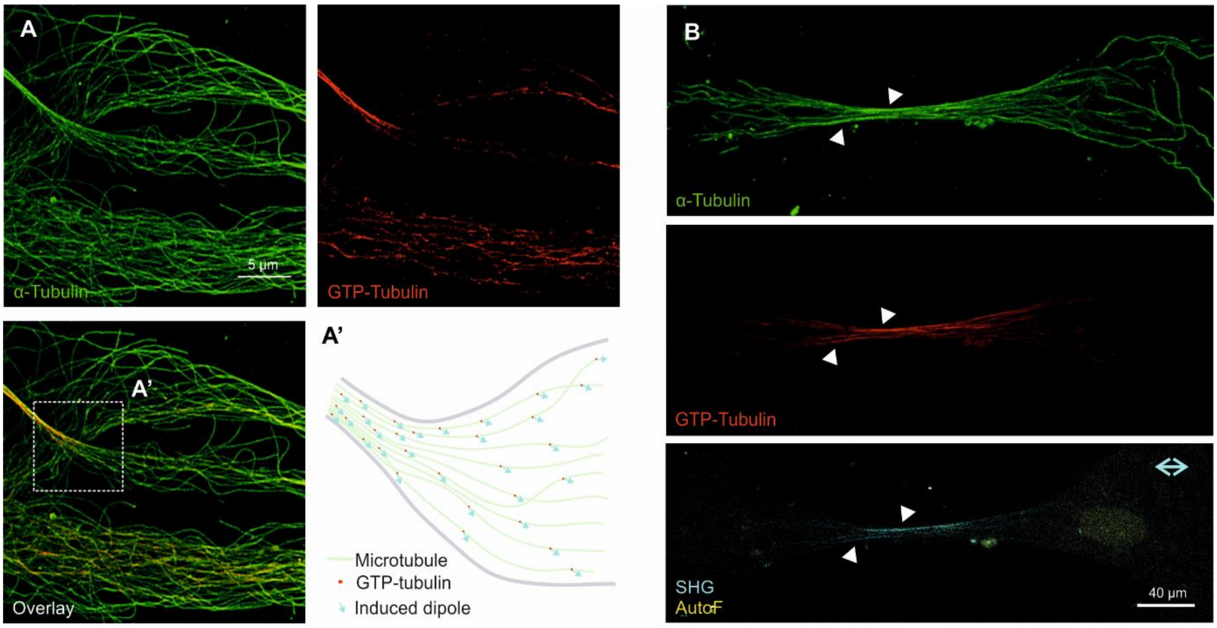

Supplementary Figure 3.

SH in fibroblasts originates from densely packed GTP-bound tubulin dimers.

(A) Staining of tubulin (α-tubulin, green) and GTP-bound tubulin (MB11, red) in fibroblasts indicating distinct GTP-tubulin sites within the microtubule lattice. (A′) Regions within the fibroblast where the cytosolic space is constricted contain many densely packed microtubules, arranging the GTP tubulin sites in a dense and parallel manner, allowing efficient constructive interference for SH imaging. (B) Microtubules in non-neuronal cells such as fibroblasts generate detectable SH signals in constricted regions (arrowheads). The SH signal in a fibroblast colocalizes with a staining for GTP-bound tubulin dimers (MB11, red) and overall microtubule staining (α-tubulin, green).

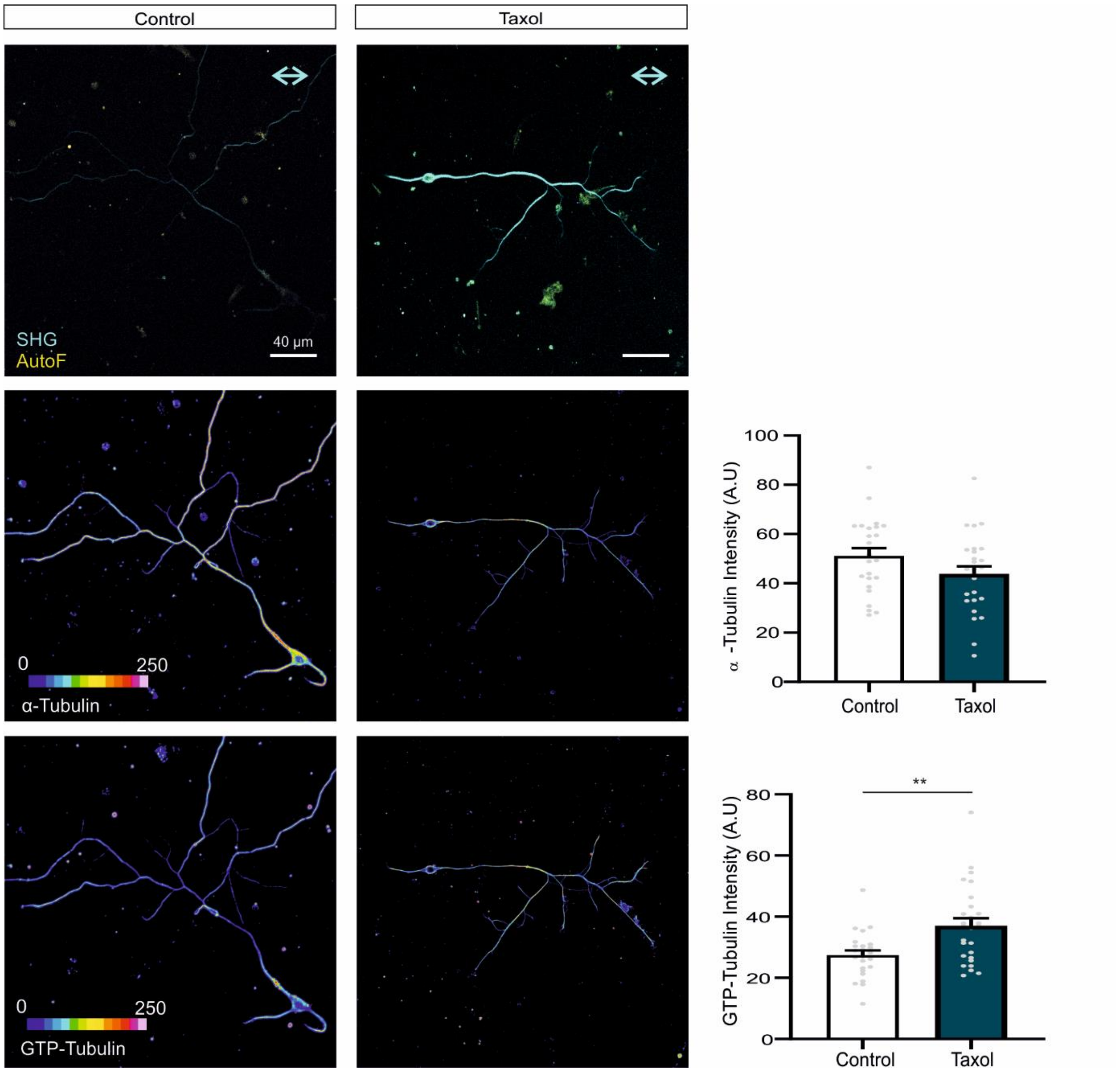

Supplementary Figure 4.

Taxol increases GTP-bound tubulin dimers and not the total amount of tubulin.

SH signals from control (DMSO) and taxol (10 nM, 4h) treated cells where taxol acts as a microtubule-stabilizing agent by increasing the amount of GTP-bound tubulin dimer conformations. The corresponding α-tubulin staining (Intensity color coded) shows no difference in total number of microtubules between both groups (n=41 biologically independent cells; $p>0.05$ Mann Whitney test). GTP-bound tubulin (MB11 ; Intensity color coded) stainings show increased fluorescence intensity in taxol treated neurons compared to controls (n=41 biologically independent cells; ** $p<0.01$ Mann Whitney test). Neuronal cultures were imaged at 7 DIV. Bar plots presented as means ± standard error of the mean. All source data are provided as a Source Data file.

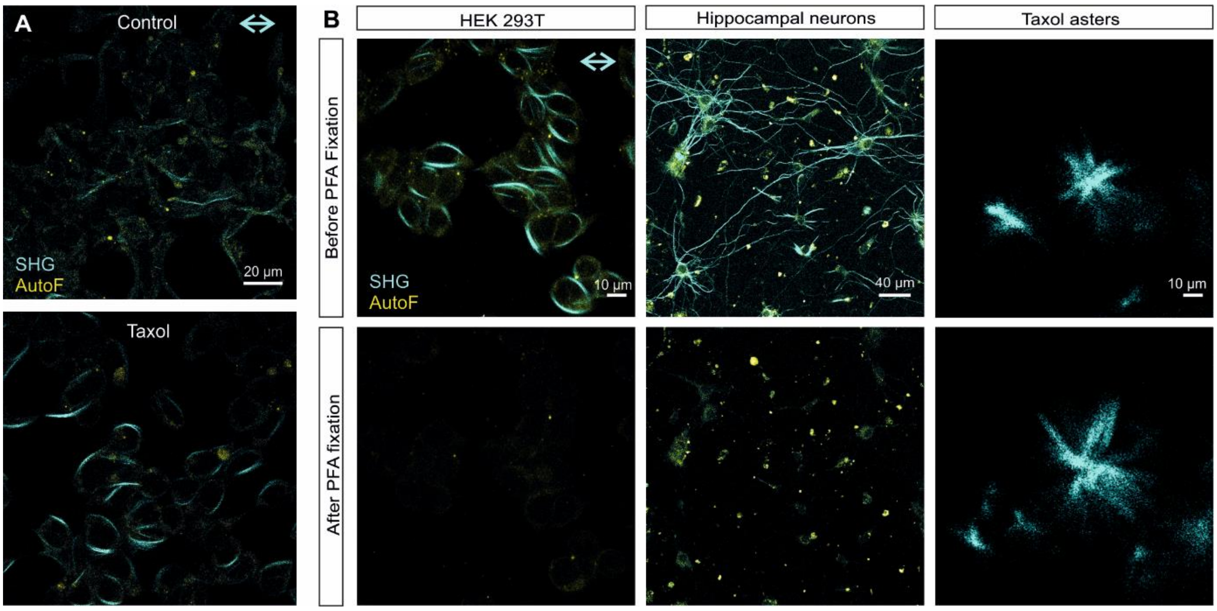

Supplementary Figure 5.

The SH signal from taxol-treated cells does not originate from taxol.

Second harmonic signals recorded in HEK 293T cells generate bright second harmonic signals upon taxol incubation, originating from tubulin and not taxol itself. (A) HEK 293T cells treated with DMSO (control) or taxol (10 nM, 4h). (B) The SH signal generated by microtubules in HEK293T cells and neuronal cultures treated with taxol (10 nM, 4h) disappears upon fixation with paraformaldehyde (PFA, 4%). As taxol is capable of generating SH signals when forming taxol asters in solution, the increase in SH signal intensity in taxol treated cells could be ascribed to the taxol molecules bound to the microtubule. Taxol asters generating SH in solution show no loss of SH signals before and after fixation (PFA, 4%). Therefore, the SH signal originating from taxol-treated cells is not related to the presence of taxol molecules whose density might be too sparse to generate a detectable signal compared to the densely packed asters formed in solution. Neuronal cultures were imaged at 7 DIV.

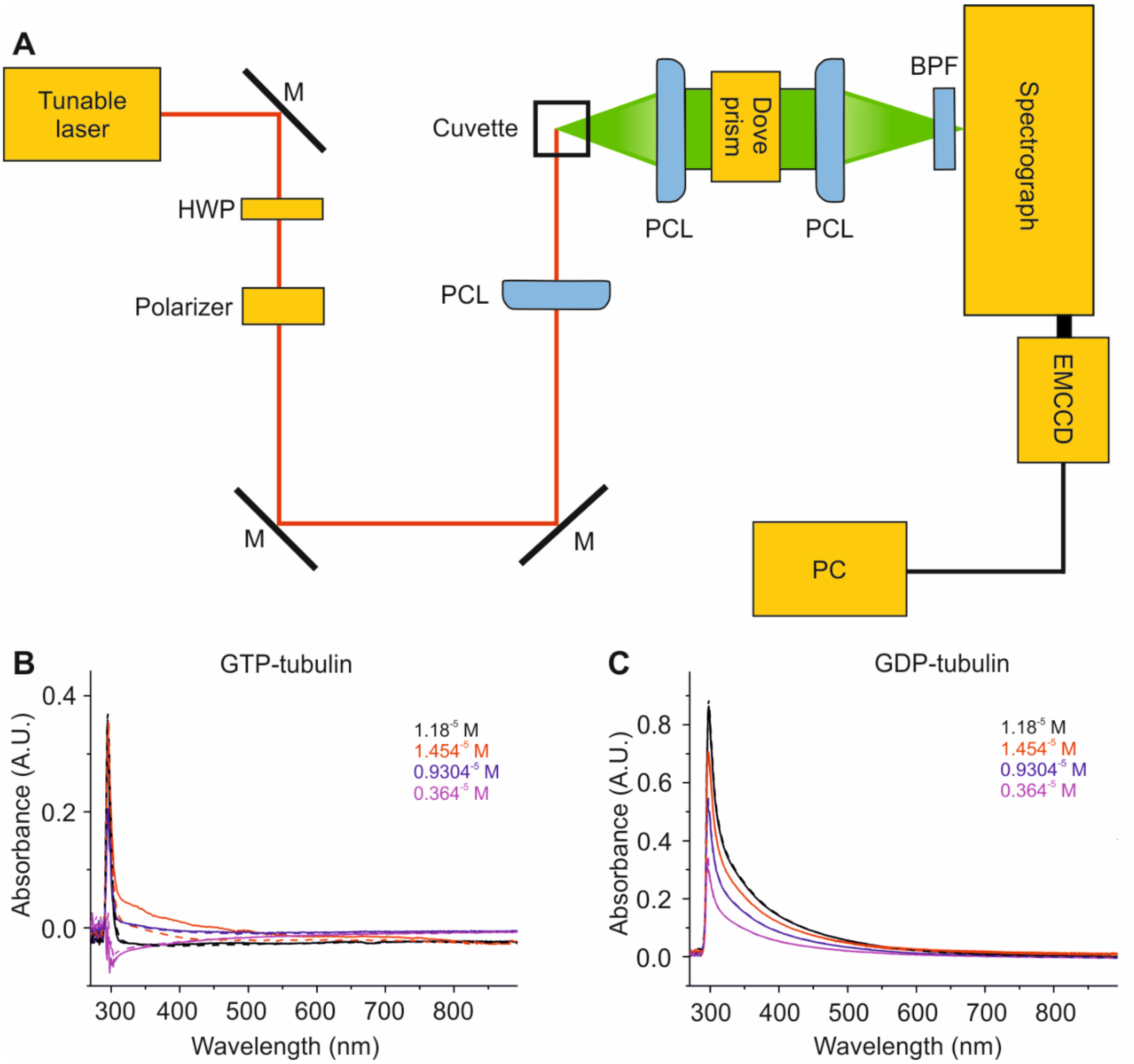

Supplementary Figure 6.

UV-VIS spectra of GTP- and GDP-bound tubulin dimers for HRS measurements.

(A) Schematic overview of the spectral HRS setup containing an Insight DS+ (Spectra-Physics) tunable femtosecond pulsed laser with an 80 MHz repetition rate and a 120 fs pulse duration; HWP, half-wave plate; PCL, plano-convex lens; M, mirror; spectrograph (Bruker IS 500); EMCCD camera (Andor Solis model iXon Ultra 897); dove prism mounted at 45° with respect to the horizontal plane; BPF, band-pass filter (FF01-890/SP-25, Semrock). (B) UV-VIS measurement of GTP- and GDP- (C) bound tubulin dimers before (solid line) and after (dotted line) HRS measurement overlap. The line colors represent the concentrations used for HRS and hyperpolarisability calculations. All source data are provided as a Source Data file.

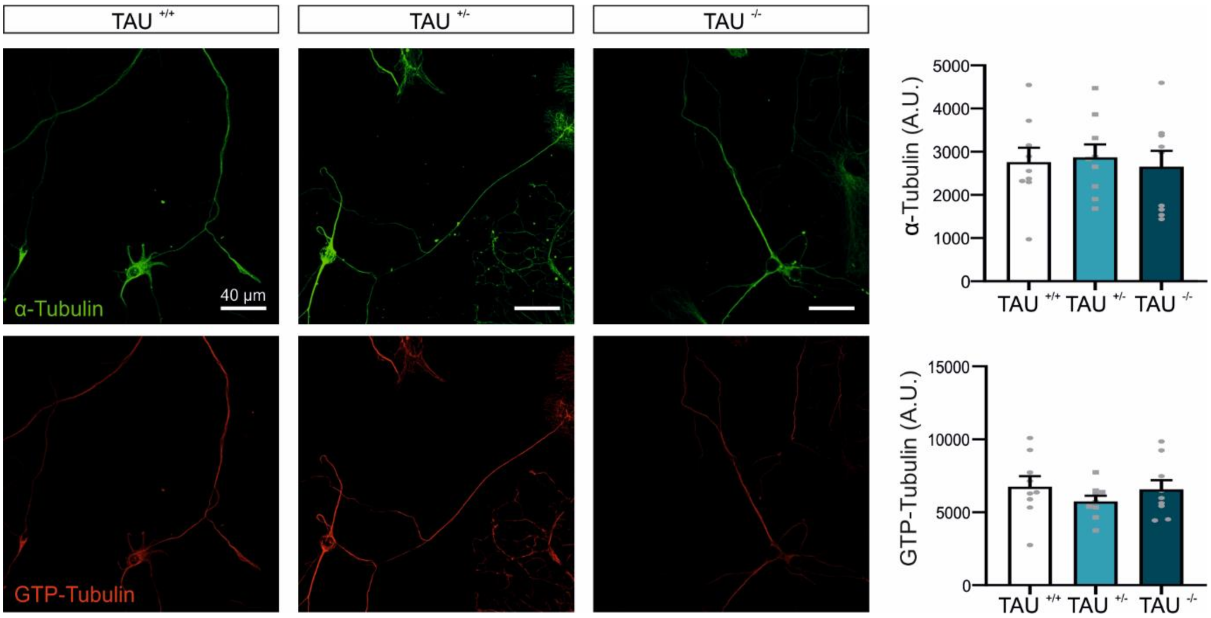

Supplementary Figure 7.

Immunohistochemistry in TAU deficient neuronal cultures shows no difference.

Wild-type ($TAU^{+/+}$), heterozygous ($TAU^{+/-}$) and knock-out ($TAU^{-/-}$) neuronal cells show no difference in total microtubule number as indicated by the fluorescence intensity of α-tubulin stainings nor a difference in the amount of stable GTP-bound tubulin dimer conformations represented by fluorescence intensity of the MB11 antibody (n=27 biologically independent cells; One-way ANOVA Dunn's multiple comparison test). Neuronal cultures were imaged at 7 DIV. Bar plots presented as means ± standard error of the mean. All source data are provided as a Source Data file.

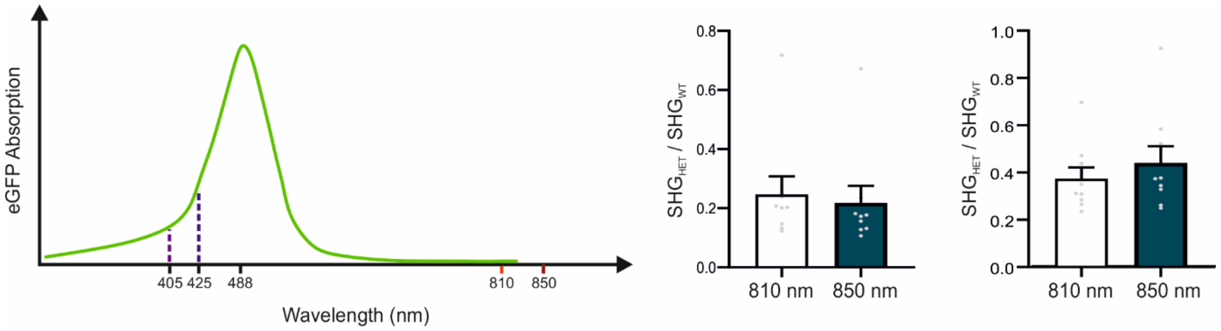

Supplementary Figure 8.

eGFP expression is not related to decreased SH intensity in TAU deficient cultures.
As the neuronal cultures prepared from TAU deficient mice have GFP-encoding cDNA into exon 1 of TAU, a GFP fusion protein (amino acids 1-31 of TAU protein) is expressed. This can be used to identify TAU deficient neurons in a neuronal culture. As our frequency doubled SH signal has a wavelength (405 nm) that borders the absorption spectrum of GFP itself, the decrease in SH signal intensity in TAU deficient cultures could theoretically be linked to absorption of our SH signal by GFP. Therefore, apart from 810 nm excitation, we also recorded SH signals in wild-type (SHG_{WT}), heterozygous (SHG_{HET}) and knock-out (SHG_{KO}) neurons using 850 nm excitation. The frequency-doubled wavelength from the red shifted excitation is located closer to the GFP absorption maximum. If absorption of our SH light by GFP would occur, more absorption, thus decreased SH signal intensity, would take place for 850 nm excitation (425 nm SH) as compared to 810 nm excitation (405 nm SHG). Comparison of SH signal ratios of heterozygous ($TAU^{+/-}$ $eGFP^{+/-}$) or knock out ($TAU^{-/-}$ $eGFP^{+/+}$) over wild-type ($TAU^{+/+}$ $eGFP^{-/-}$) cells show no difference between 810 or 850nm excitation (n=27 biologically independent cells; $p>0.05$ Mann Whitney test). As no decrease in the ratio was found from 810 to 850 nm excitation, we can exclude the absorptive effect of GFP as the reason for decreased SH signal intensities in TAU deficient cultures. Neuronal cultures were imaged at 7 DIV. Bar plots presented as means ± standard error of the mean. All source data are provided as a Source Data file.
